# Effect of Internal Pores Formed by a Superabsorbent Polymer on Durability and Drying Shrinkage of Concrete Specimens

**DOI:** 10.3390/ma14185199

**Published:** 2021-09-10

**Authors:** Il-Sun Kim, So-Yeong Choi, Yoon-Suk Choi, Eun-Ik Yang

**Affiliations:** 1 Research Institute for Disaster Prevention, Gangneung-Wonju National University, Jukheon-gil 7, Gangneung-si 25457, Gangwon-do, Korea; iskim@gwnu.ac.kr (I.-S.K.); csy7510@gwnu.ac.kr (S.-Y.C.); 2 Construction Technology Research Center, Korea Conformity Laboratories, Gasan Digital 1-ro, Geumcheon-gu, Seoul 08503, Korea; yoons0305@kcl.re.kr; 3 Department of Civil Engineering, Gangneung-Wonju National University, Jukheon-gil 7, Gangneung-si 25457, Gangwon-do, Korea

**Keywords:** superabsorbent polymer, compressive strength, drying shrinkage, pore size distribution, chloride penetration depth, durability

## Abstract

In this study, the effect of internal pores formed by a superabsorbent polymer (SAP) was analyzed by evaluating the compressive strength, chloride penetration depth, drying shrinkage, and pore size distribution of SAP-containing concrete, while securing workability using a water-reducing agent (WRA). The experimental results showed that the amount of WRA necessary increased as the amount of SAP added increased, and that the compressive strength was the highest when the SAP content was 1.5% of the concrete mix. Drying shrinkage tended to decrease as the SAP content increased, and it decreased by approximately 31–41% when the SAP content was 2.0% compared to that of the reference mix. The SAP expanded by approximately three times inside concrete, and it was distributed within the internal pores of air-entrained concrete. The optimal SAP content in concrete mix was 1.5%, and an SAP content of 2.0% or higher adversely affected the workability and compressive strength.

## 1. Introduction

Concrete is undoubtedly an excellent civil and building material. Many researchers are working on various admixtures to improve the performance of concrete. Fly ash and blast furnace slag are widely known admixture materials to replace cement [1,2,3], and some researchers used industrial wastes such as waste glass and steel slag to replace aggregates [4,5,6]. Recently, studies on materials that can improve the performance of cement composites by using small amounts are being conducted [7,8,9,10]. One of them is the C-S-H seeds. Since the addition of fly ash significantly retards the hydration process of the paste, the initial strength (within about 24 h) can be increased by adding C-S-H seeds [7]. The other is a superabsorbent polymer (SAP). An SAP is a polymer compound that can absorb and store a large amount of water. It is known that SAPs can absorb approximately 100–400 times their weight in water and they are manufactured in various sizes and shapes [9,10]. SAPs expand inside fresh concrete by absorbing mixing water and they contract in a dry environment by releasing moisture. In this case, the released water supplies moisture to the concrete, creating an internal curing effect. As a result of the internal curing effect, the durability of concrete improves owing to reduced shrinkage and a greater strength [9,11]. Since internal curing by SAPs can significantly reduce autogenous shrinkage and improve concrete strength, many studies have been conducted on this subject [12,13,14,15,16,17,18].

SAPs decrease workability by absorbing mixing water, and workability rapidly decreases as the SAP content increases [19]. In addition, owing to the absorption of mixing water by the SAP, the actual W/C ratio (effective W/C ratio) becomes lower than the design W/C ratio. Therefore, it is necessary to secure the design W/C ratio and workability by adding mixing water in proportion to the amount absorbed by the SAP. In fact, various experiments have been performed in which mixing water was added to the concrete mix [9,10,11,12,13,14,15,20,21,22,23,24,25,26,27]. Adding mixing water, however, increases the unit water content and eventually increases the amount of moisture in the concrete, including the moisture absorbed by the SAP. The increased moisture is expected to offset the positive effects of the addition of SAPs, such as the internal curing effect. Therefore, the increased moisture adversely affects the durability and performance of concrete. As a consequence, research on methods to secure workability without adding mixing water is required.

Drying shrinkage is a phenomenon that occurs when the moisture in concrete is released owing to low outside humidity. As moisture diffuses from the concrete surface to the outside after hardening, the capillary pressure, disjoining pressure, and surface tension change, which causes drying shrinkage. In general, drying shrinkage is significantly affected by the W/C ratio, and it tends to decrease as the W/C ratio decreases. It is well known that the addition of SAPs decreases autogenous shrinkage, and many studies have been conducted on this subject [12,14,16,18,28]. However, studies on the drying shrinkage of SAP concrete are relatively scarce. As the addition of SAPs decreases the design W/C ratio, it can also affect the drying shrinkage. In previous studies [14,21,26], the drying shrinkage of concrete mixes with SAPs was measured while additional mixing water was also added. Thus, it is difficult to evaluate the effects of only adding an SAP to the concrete mix. Therefore, it is necessary to conduct research on the effect of SAP addition by measuring the drying shrinkage of concrete with no additional mixing water.

The SAP that absorbs moisture inside the concrete mix expands, and its diameter increases by approximately three times according to a previous study [29]. This volume acts as a pore after hardening. Therefore, it is expected that the pore sizes inside concrete can be adjusted by changing the size of the dry SAP used. Pores inside concrete are closely related to freeze-thaw resistance and durability. Thus, if the pore size can be adjusted, it will significantly contribute to an improvement in concrete performance. Therefore, it is necessary to analyze the pore size changes inside concrete due to SAPs by analyzing the pore size distribution in concrete mixed with SAPs.

In this study, a water-reducing agent (WRA) was used to prevent a decrease in workability caused by the addition of SAPs and to exclude the influence of adding mixing water to secure workability. In addition, the effect of internal pores formed by SAPs was investigated by evaluating the slump, air content, compressive strength, chloride penetration depth, drying shrinkage, and pore size distribution of concrete mixed with an SAP or an air-entraining (AE) agent. At this time, water-curing and sealed-curing experiments were separately conducted to evaluate the internal curing effect. 

## 2. Experimental Method

### 2.1. Test Plan

In this study, the slump, air content, compressive strength, chloride penetration depth, drying shrinkage, and pore size distribution were measured on concrete that included an added SAP. The results were compared to the corresponding values in specimens without added SAP in order to evaluate changes in the properties of concrete. The SAP used in this study was atypical, the W/C ratio was set to 40% or 50%, and the amount of SAP added 0 (Ref.), 1.0%, 1.5%, and 2.0% of the cement mass. Additionally, a case in which an AE agent was added instead of an SAP was also studied.

The concrete was cured under water or in sealed curing conditions to evaluate the internal curing effect of the SAP. Water curing was conducted by immersing the specimens in a constant-temperature water bath (20 ± 3 °C, RH 100%) after de-molding. Sealed curing was performed by sealing specimens with plastic material and storing them in a constant temperature and humidity chamber (20 ± 3 °C, RH 60%) after de-molding, so that moisture transfer to and from the outside could be restricted as much as possible. Table 1 and Table 2 list the variables and mix proportions used in the experiments.

### 2.2. Materials

**Figure 1:**
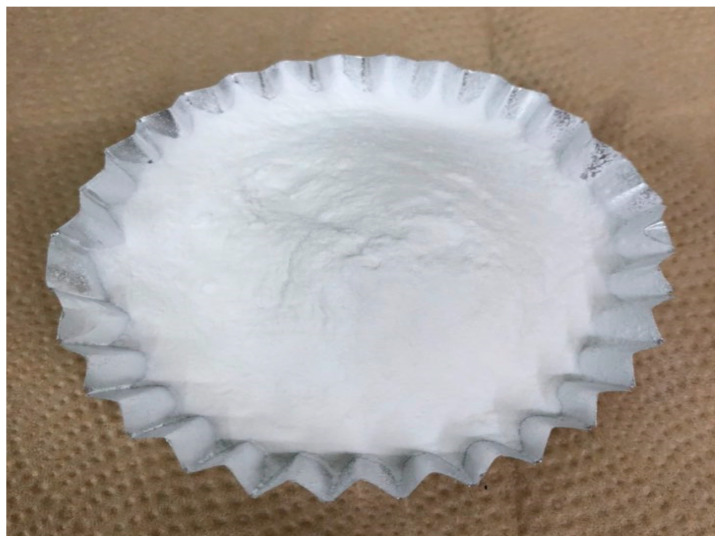
Superabsorbent polymer (SAP).

The SAP used in this study was an acrylic acid (AA)-based product, which is atypical. The shape of the SAP was determined by the manufacturing method. An atypical SAP product is manufactured in a large lump by gel polymerization and then crushed to the desired particle size. The SAP used in this study had particle sizes ranging from 38 to 100 μm. Figure 1 illustrates the SAP used. The moisture absorption time and absorptivity of SAP depends on its particle size, and the absorptivity varies depending on the pH. The absorptivity of SAP affects the workability and characteristics of concrete. The maximum water absorptivity of SAPs obtained from a previous study [19] is shown in Table 3. The time required to reach the maximum absorptivity (saturation state) of the SAP was less than 3 min.

The cement used in this study was ordinary Portland cement (ASTM C 150) [30], and its physical and chemical properties are listed in Table 4. 

The coarse aggregate used was a crushed stone with a maximum size of 19 mm, and the fine aggregate used was sand with a maximum size of 5 mm. Table 5 lists the physical properties of the aggregates used.

The admixtures used in this study were an AE agent and a water-reducing agent (WRA). A polycarboxylic acid-based water-reducing agent was used.

### 2.3. Test Methods

#### 2.3.1. Properties of Fresh Concrete

To evaluate the properties of fresh concrete mixed with the SAP, the slump and air content were measured in accordance with ASTM C 143 [31] and ASTM C 231 [32], respectively. In addition, the slump and air content were measured simultaneously after mixing to minimize the influence of the absorptivity of the SAP.

In most previous studies that used SAPs [16,20,21,22,23], tests were performed by adding mixing water in proportion to the absorptivity caused by the SAP content. In this study, however, WRA was used without the use of additional mixing water to secure workability. The target slump (120~180 mm) was set, and the WRA content was increased according to the SAP content so that the target slump could be reached for all concrete mixes.

#### 2.3.2. Compressive Strength

**Figure 2:**
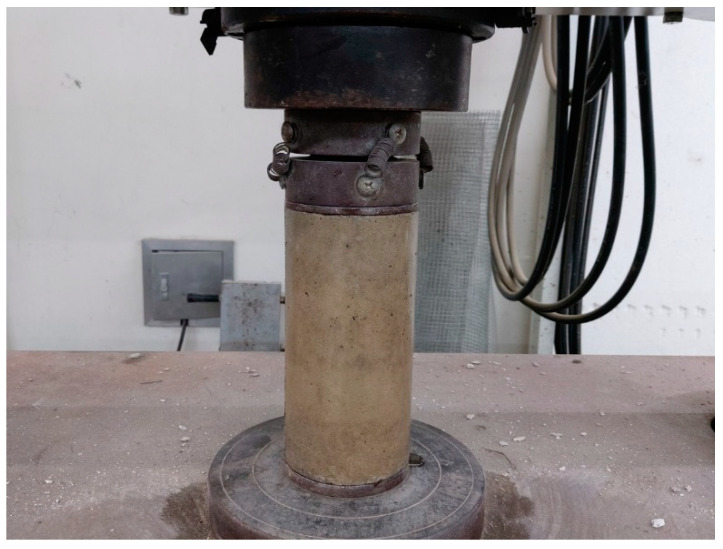
Compressive strength test configuration.

In this study, the compressive strength of concrete was measured using Ø100 mm × 200 mm cylinder specimens in accordance with ASTM C 39 [33]. The specimens were cured for 28 and 91 days for each curing method, and their compressive strengths were measured using a universal testing machine (Company S, 1000 kN). The average compressive strengths of three specimens was used as the final value. Figure 2 shows the compressive strength test configuration.

#### 2.3.3. Chloride Penetration Depth

**Figure 3:**
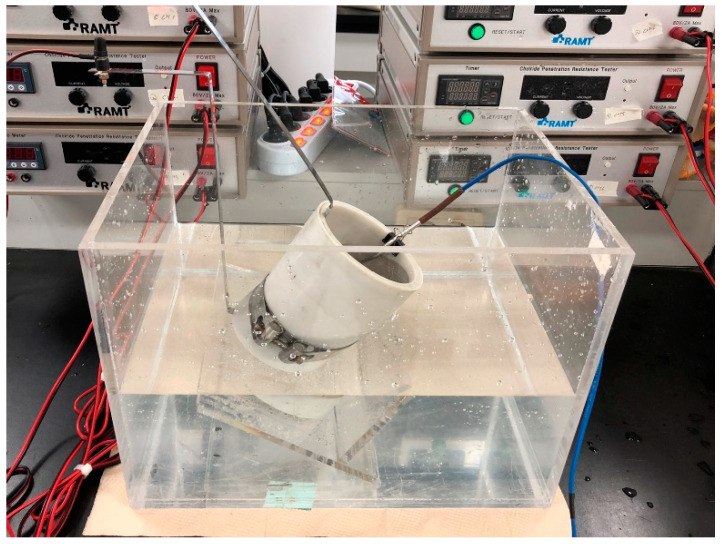
Chloride penetration resistance.

A rapid chloride penetration test was conducted to evaluate the chloride penetration resistance with the addition of the SAP. The rapid chloride penetration test was performed according to the NT Build 492 standard. A concrete specimen with a size of Ø100 mm × 200 mm was cured for 28 days according to the curing method, and then cut into 50 ± 2 mm sections for testing. Subsequently each cut specimen was immersed in water for at least 24 h before a chloride penetration test, to prevent instantaneous absorption according to the internal moisture state. A 0.3 N sodium hydroxide (NaOH) aqueous solution was added to the positive electrode (+) of the test body, a 3% sodium chloride (NaCl) aqueous solution was added to the negative electrode (−), and a voltage of 30 V was applied for 8 h. The specimens for which the rapid chloride penetration test was completed were split, and the chloride penetration depth was measured by the silver nitrate discoloration method. The average value of three specimens was used. Figure 3 shows the configuration of the chloride penetration resistance test (NT-Build 492).

#### 2.3.4. Drying Shrinkage

**Figure 4:**
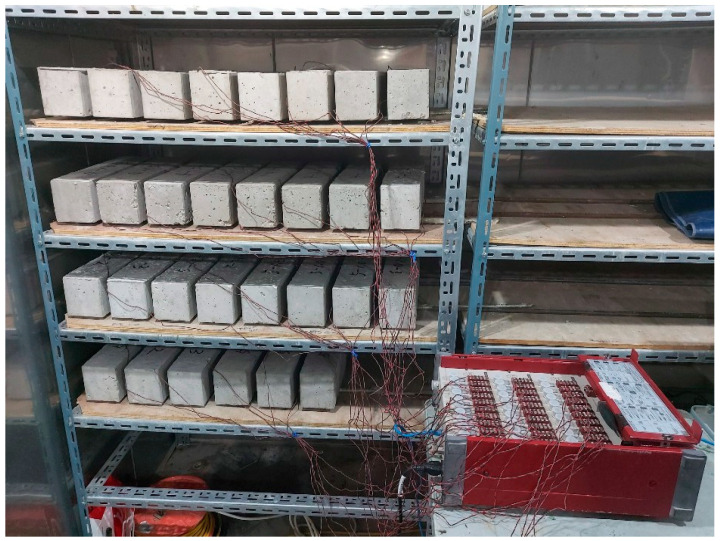
Drying shrinkage test.

In this study, a mold gauge was used to measure the drying shrinkage according to the SAP content. The mold gauge is suitable for measuring the internal strain as it is embedded in concrete, and a PMF type was used. Prismatic specimens of size 100 mm × 100 mm × 400 mm were prepared in accordance with ASTM C 157 [34] for use in the drying shrinkage test. The mold gauge was fixed at the center of the acrylic mold, and concrete was poured. The concrete was cured for 24 h and then de-molded. Water curing was then conducted for six days. The specimens were moved into a constant temperature and humidity chamber (20 ± 3 °C, RH 60%), and the drying shrinkage was measured using a data logger (Company T, 30 ch). Figure 4 shows the configuration of the drying shrinkage test. 

#### 2.3.5. Pore Size Distribution

**Figure 5:**
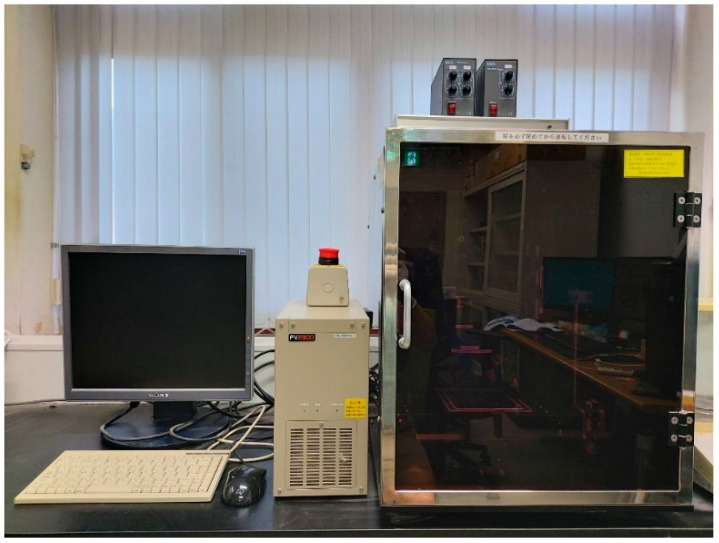
Instrument of image analysis method.

In this study, the entrained air and pore sizes in concrete were measured using image analysis and applying the traverse method in accordance with ASTM C 457 [35]. The measuring instrument used in this study was an optical microscope that can magnify polished specimens 50~120 times, and a resolution of 0.00899 mm/pix was used. The total length of the traverse line was 2320 mm, and 63 images per specimen were used for measurement. For the pore structure analysis, Ø100 mm × 200 mm specimens were cut into samples with a thickness of 50 mm, and the sample surface was polished for measurement. The average measurement values of three specimens were used. Figure 5 shows the instruments used for image analysis.

## 3. Results and Discussion

### 3.1. Properties of Fresh Concrete

#### 3.1.1. Slump Value

**Figure 6:**
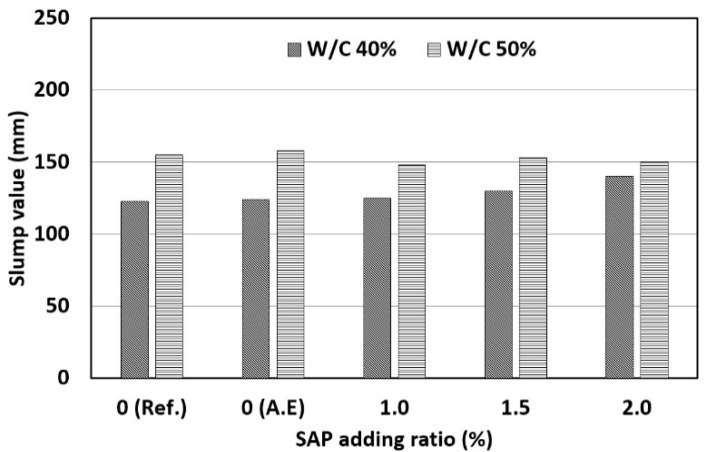
Results of slump value.

The results of measuring the slump of concrete mixed with the SAP according to the W/C ratio and SAP content are shown in Figure 6. The test results show that all the concrete samples reached the target slump (120~180 mm) set in this study, and similar slump values were observed for the studied W/C ratios. This was an expected result because different amounts of WRA were used for each concrete specimen so that the target slump could be reached. In a previous study [19], the slump value decreased as the content of SAP increased. The slump value reduction due to the absorption of mixing water by SAPs is already well known.

Table 6 shows the slump values according to the WRA ratio. In this study, the WRA ratio, necessary to obtain the desired slump value, increased as the added SAP ratio increased. The WRA ratio showed a tendency to linearly increase until the SAP ratio reached 1.5%. As the SAP ratio increased by 0.1%, the WRA ratio had to be increased by 0.04%. When the SAP adding ratio was 2.0%, the WRA ratio necessary to achieve the target slump value ranged from 2.0% to 3.0%, which exceeded the general WRA ratio (1.0% or less). In particular, when the SAP ratio increased from 1.5% to 2.0%, the WRA ratio necessary increased rapidly. This indicates that the SAP ratio must be 1.5% or less of the cement mass to secure workability using the WRA.

#### 3.1.2. Air Content

**Figure 7:**
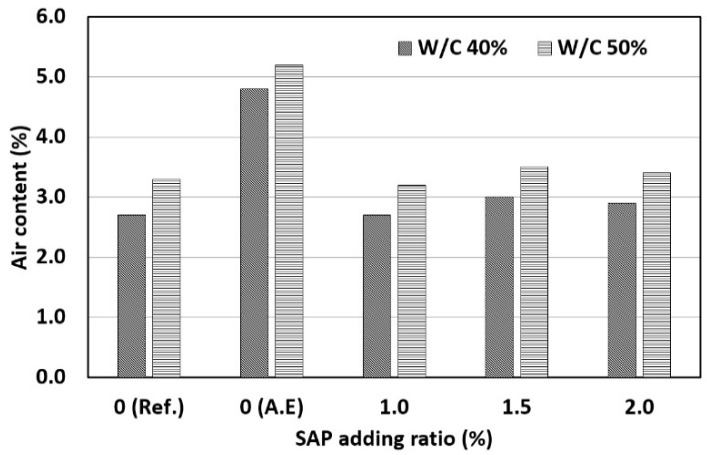
Results of air content.

The results of measuring the air content of concrete mixed with SAPs according to the W/C ratio and SAP ratio are shown in Figure 7. The test results showed that there was no significant difference in the air content depending on the SAP ratio. However, when the AE agent was added the air content tended to increase owing to the AE effect. It appears that even though the SAP expanded inside the concrete, it did not have a significant influence on the air content of fresh concrete.

The air content of hardened concrete mixed with SAPs may increase [36]. In fresh concrete, however, this tendency cannot be accurately measured by air content measurements using the pressure method [19]. Therefore, it is judged that the accurate air content of concrete mixed with SAP must be measured using hardened concrete.

### 3.2. Compressive Strength

**Figure 8:**
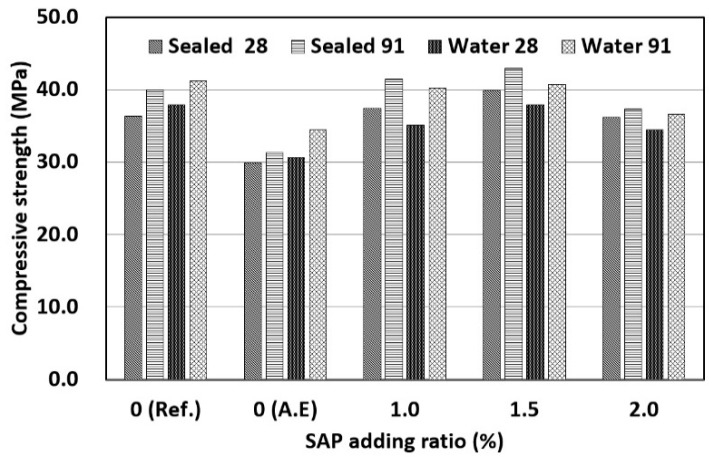
Results of compressive strength (W/C 40%).

The results of measuring the compressive strength of concrete mixed with SAPs according to the W/C ratio are shown in Figure 8. First, when the W/C ratio was 40%, there was no regular trend due to the addition of the SAP. In the case of water curing, the reference mix (Ref.) exhibited the highest compressive strength. The same compressive strength as that of the reference mix was observed when the SAP content was 1.5%. For water curing, the internal curing effect by the drying of the SAP cannot be expected because the inside and outside of concrete were maintained under wet conditions [19]. In addition, the compressive strength is affected by the strength increase due to a reduction in the effective W/C ratio caused by the water absorption characteristics of SAP and the strength decrease due to the increase in SAP pores. In conclusion, the compressive strengths was restored at an SAP content of 1.5% because the strength increase due to the reduced W/C ratio was dominant. The compressive strength decreased when the SAP content was 2.0%, compared with the strength of the specimen with 1.5% SAP content. This appears to be because the influence of the SAP pores was higher than that of the effective W/C ratio. This indicates that the maximum SAP content for water curing based on the compressive strength was 1.5%. In the case of sealed curing, the highest compressive strength was observed when the SAP content was 1.5%. When the SAP content was 1.0% or higher, sealed curing led to a higher compressive strength than water curing. In particular, when the SAP content was 1.5%, sealed curing exhibited a higher compressive strength than that achieved by water curing of the reference mix (Ref.), which is the most favorable curing method for the development of concrete strength. This is because the compressive strength was increased by the reduction in the effective W/C ratio and the internal curing effect of the SAP. Therefore, an appropriate SAP content can increase the compressive strength without the need for water curing owing to the internal curing effect. When the SAP content was 2.0%, the compressive strength decreased compared to that when the SAP content was 1.5%. This is because an increase in SAP content increased the internal curing effect as well as the number of SAP pores and an SAP content of 2.0% or higher made the influence of SAP pores more dominant. Therefore, in the case of sealed curing, the optimal SAP content for an increase in compressive strength was determined to be 1.5%. 

**Figure 9:**
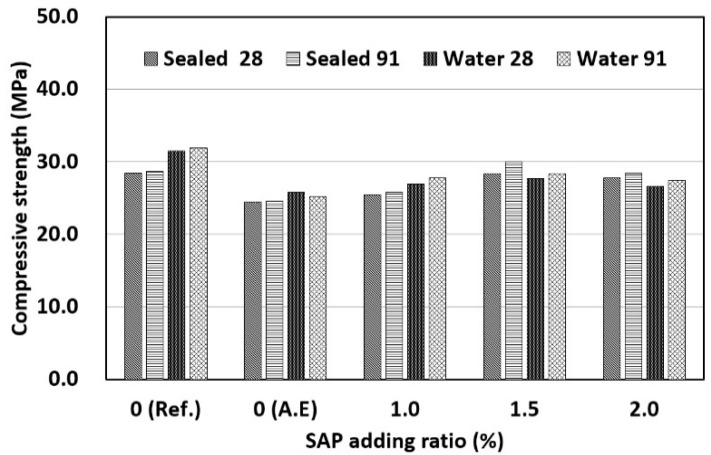
Results of compressive strength (W/C 50%).

When the W/C ratio was 50% (Figure 9), the reference mix (Ref.) exhibited the highest compressive strengths regardless of the curing conditions. First, in the case of water curing with the addition of SAP, the highest compressive strength was observed when the SAP content was 1.5%, but there was no significant difference from those at other SAP contents. It appears that the compressive strength was affected by the absence of the internal curing effect and the offset between the effective W/C ratio and SAP pores in the case of water curing. In the case of sealed curing, the highest compressive strength was observed when the SAP content was 1.5%. When the SAP content was 1.5% or higher, sealed curing exhibited a higher compressive strength than that observed after water curing. This appears to be due to the effective W/C ratio and the internal curing effect. In addition, when the SAP content was 2.0%, the compressive strength decreased compared to the strength when the SAP content was 1.5%. This is the same result as when the W/C ratio was 40%. In summary, the optimal SAP content at a W/C ratio of 50% was determined to be 1.5%. When the W/C ratio was 40% or 50%, sealed curing tended to produce a higher compressive strengths than water curing according to the SAP content. As the W/C ratio decreased, higher compressive strengths were observed even at low SAP contents. This indicates that the influence of SAP is higher when the W/C ratio is 40%. In a previous study [9], it was reported that the reduction in concrete strength because of pores is dominant when the W/C ratio is 45% or higher because of the insignificant influence of the internal curing effect and that strength improvement can be expected when the W/C ratio is lower than 45%. In this study, increase in strength was also greater when the W/C ratio was 40%, owing to the internal curing effect of SAP.

When the AE agent was used, the lowest compressive strength was observed regardless of the W/C ratio. The use of the AE agent may improve workability owing to the entrained air, but it may decrease the compressive strength owing to the increase in air content. Therefore, it appears that the addition of an AE agent decreases the compressive strength.

### 3.3. Chloride Penetration Depth

**Figure 10:**
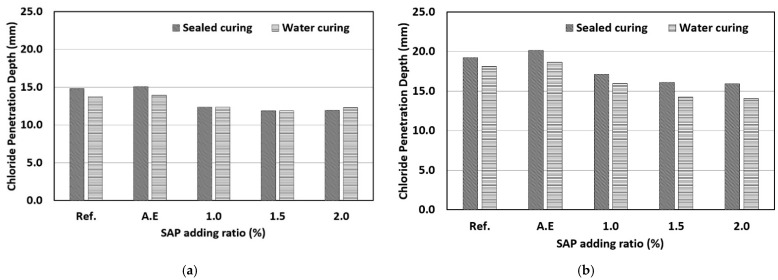
Results of chloride penetration depth: (**a**) Water–cement ratio 40%; (**b**) Water–cement ratio 50%.

The results of the chloride penetration depth measurement by the rapid chloride penetration resistance test are shown in Figure 10. First, in the case of a W/C ratio of 40% (Figure 10a), the mix with SAP showed a lower penetration depth than that of the reference mix (Ref.). It was determined that the addition of the SAP reduced the effective W/C ratio and improved the water tightness through the internal curing effect, thereby increasing the chloride penetration resistance. The addition of SAPs has a positive effect of reducing the effective water–cement ratio and the internal curing but it also has a negative effect of increasing internal voids. Due to the conflict between these two effects, the optimal addition ratio was determined to be 1.0%, and even if the addition ratio was increased, there was no significant effect on the chloride penetration resistance.

In the case of a W/C ratio of 50% (Figure 10b), the mix with the SAP still showed a lower penetration depth than that of the reference mix (Ref.). It was determined that the addition of the SAP reduced the effective W/C ratio, and improved water tightness through the internal curing effect, thereby increasing the chloride penetration resistance. All SAP mixes with a W/C ratio of 50% led to a lower penetration depth after water curing. As the effect of the SAP on the internal curing was not substantial, the effect of improving the chloride penetration resistance under sealed curing was not observed with a W/C ratio of 50%.

### 3.4. Drying Shrinkage

**Figure 11:**
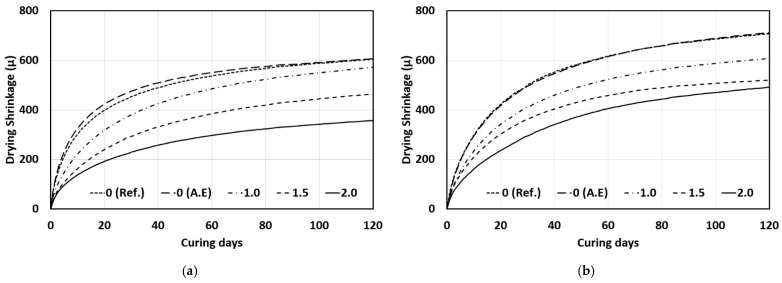
Results of drying shrinkage: (**a**) Water-cement ratio 40%; (**b**) Water-cement ratio 50%.

The results of measuring the drying shrinkage of concrete mixed with the SAP according to the W/C ratio are shown in Figure 11. The drying-shrinkage measurement results showed that the drying shrinkage decreased as the W/C ratio decreased, and that the drying shrinkage tended to decrease as the SAP content increased [27,37,38]. In general, the drying shrinkage was significantly affected by the W/C ratio, and it tended to decrease as the W/C ratio decreased. In addition, when the SAP content was 2.0%, the drying shrinkage decreased by approximately 41% at a W/C ratio of 40% and by approximately 31% at a W/C ratio of 50% compared to that of the reference mix (Ref.). The addition of the SAP significantly decreased the drying shrinkage, regardless of the W/C ratio.

In this study, mixing water was not added according to the SAP content. Therefore, the same unit water content was used in all concrete mixes under the same W/C ratio. Drying shrinkage occurs when the moisture in concrete is released to the outside. Therefore, a similar drying shrinkage should occur for concrete mixes with the same unit water content if the same materials are used. However, as the SAP content increased, the drying shrinkage decreased significantly. This appears to be due to the influence of the SAP which can be mainly divided into three categories.

The first category is the influence of the reduced effective W/C ratio. As the SAP content increased, the effective W/C ratio decreased owing to the water absorption of the SAP, and thus, the drying shrinkage decreased. The second category is the influence of the reduced autogenous shrinkage. The drying shrinkage measured in this study includes autogenous shrinkage. The addition of the SAP significantly decreased autogenous shrinkage because of the internal curing effect in concrete, and the rate of decrease increased as the SAP content increased. Therefore, the reduced autogenous shrinkage by the SAP appears to have affected the decrease in drying shrinkage. The third category is the influence of relative humidity inside the concrete. The SAP added to the concrete expanded by absorbing water and maintained its saturation state during the water-curing period. When drying-shrinkage measurements were conducted under constant temperature and humidity conditions (20 °C, RH 60%) upon the completion of water curing, shrinkage occurred as moisture evaporated from the concrete surface. Inside our sample concrete, however, a high relative humidity was maintained by the SAP in the saturation state, and the relatively high humidity could be maintained for longer with higher SAP content. The internal humidity appeared to decrease the drying shrinkage by interfering with the drying of concrete and the rapid evaporation of moisture.

In some studies [21,26], the drying shrinkage of mortar mixed with an SAP showed a tendency to increase as the SAP content increased, which is contrary to the results of this study. In previous studies [21,26], the SAP content was lower than 0.9% and mixing water was added according to the SAP content considering the water absorptivity of the SAP. With the addition of water, the total water-binder ratio increased as the SAP content increased. Therefore, the effective W/C ratio did not decrease, and it appears that the drying shrinkage showed a tendency to increase as the unit water content inside the concrete increased. It also appears that a different tendency from that observed in the studies above [21,26] occurred in this study because mixing water was not added according to the SAP content.

### 3.5. Pore Size Distribution

**Figure 12:**
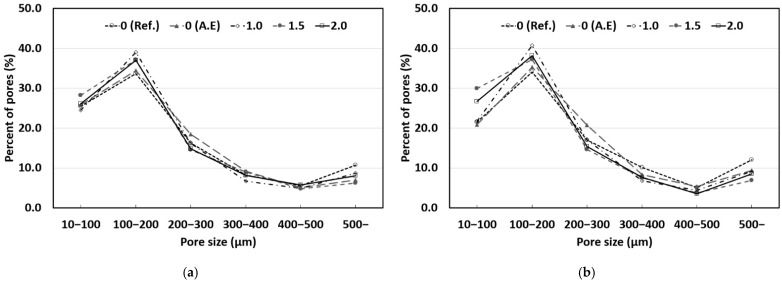
Results of pore size distribution (W/C 40%): (**a**) Sealed curing; (**b**) Water curing.

**Figure 13:**
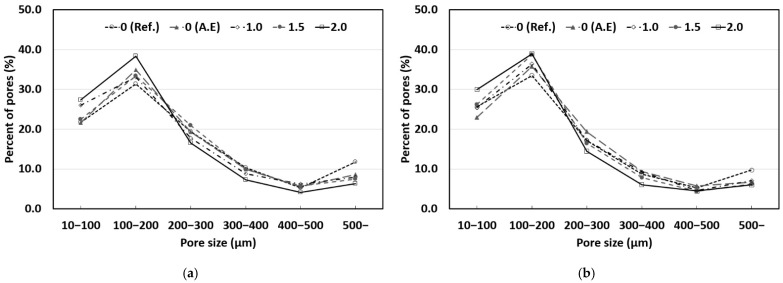
Results of pore size distribution (W/C 50%): (**a**) Sealed curing; (**b**) Water curing.

The macroscopic pore size distributions of concrete mixed with SAP according to the W/C ratio are shown in Figure 12 and Figure 13. The X-axis of the graphs indicates the pore size, and the Y-axis specifies the ratio of the pore size to the total number of pores.

In general, entrained air pockets are known to have a size between 50 and 300 μm. In a previous study [29], it was reported that the diameter of the expanded SAP was three times larger than that of the dry SAP. Therefore, it can be expected that the SAP used in this study will expand to sizes between 100 and 300 μm inside concrete and that it will have sizes similar to those of entrained air. Table 7 shows the pore size distribution measurement results, divided based on a pore size of 300 μm. As shown in Table 7, the addition of SAP and the AE agent caused pores smaller than 300 μm to increase in all the concrete mixtures compared to those in the reference mix (Ref.). It appears that pores at the entrained air level increased owing to the air entrainment effect of the AE agent and the expansion of the SAP. It is expected that desired pore sizes inside concrete can be obtained by adjusting the size of the dry SAP used.

The reference mix (Ref.) exhibited a relatively higher proportion of pores larger than 300 μm than those in other concrete mixes. This appears to be because a large number of relatively large pores (500 μm or larger) and entrapped air were distributed with the general pore size distribution of concrete. It is expected that the addition of the AE agent and an SAP can improve the durability of concrete by decreasing the proportion of large pores (300 μm or larger), which may have an adverse effect on the durability of concrete.

## 4. Conclusions

In this study, the effect of internal pores formed by an SAP on the mechanical properties and drying shrinkage of concrete was evaluated, and the results are summarized as follows:

1. As SAP content increased, the WRA content required to secure workability increased. The maximum SAP content to secure workability was determined to be 1.5% or less.
2. For the compressive strength of concrete mixed with SAP, the highest compressive strength was observed when the SAP content was 1.5% regardless of the curing method. When the SAP content was 1.5% or higher, sealed-cured specimens exhibited a higher compressive strength than water-cured specimens. This indicates that the compressive strength was affected by a reduction in the effective W/C ratio and the internal curing effect.
3. The chloride penetration depth decreased when SAP was added, compared to that of the reference mix. The chloride penetration resistance was judged to improve owing to a decrease in the effective W/C ratio and the internal curing effect of SAP.
4. As the SAP content increased, drying shrinkage showed a tendency to decrease. When the SAP content was 2.0%, the drying shrinkage decreased by approximately 31~41% compared to that of the reference mix depending on the W/C ratio. It appears that the drying shrinkage decreased owing to the combined effect of the reduction in the effective W/C ratio, the reduction in autogenous shrinkage, and the influence of the humidity inside the concrete.
5. The addition of SAP increased the number of pores smaller than 300 μm compared to those in the reference mix. It is expected that desired pore sizes can be obtained inside concrete by adjusting the size of the dry SAP.

## Figures and Tables

**Table 1:** Test variables.

Conditions	Variables		
	Compressive Strength	Drying Shrinkage	Chloride Penetration Depth Pore Distribution
W/C (%)	40, 50	40, 50	40, 50
SAP-adding ratio (C×%)	0 (Ref.), 0 (A.E), 1.0, 1.5, 2.0		
Specimen size (mm)	Ø100 × 200	100 × 100 × 400	Ø100 × 50
Curing condition (20 ± 3 °C)	Water, Sealed	Water	Water, Sealed
Curing days	28, 91	7	28

**Table 2:** Mix proportions of concrete.

W/C (%)	SAP (C×%)	Unit Weight (kg/m^3^)				SAP (kg)	AE (C×%)	WRA (C×%)
		W	C	S	G			
	0 (Ref.)	170	425	721	985	-	-	0.4
	0 (AE)					-	0.005	0.4
	1.0					4.250	-	0.8
	1.5					6.375	-	1.0
	2.0					8.500	-	3.0
50	0 (Ref.)	173	346	781	983	-	-	0.3
	0 (AE)					-	0.005	0.3
	1.0					3.460	-	0.7
	1.5					5.190	-	0.9
	2.0					6.920	-	2.0

AE: Air entraining agent, WRA: water reducing agent.

**Table 3:** Water absorptivity of the SAP.

Type	pH 7	pH 11	pH 13	CFW
Absorptivity (g/g)	112.7	32.9	25.7	19.6

CFW: Cement filtered water.

**Table 4:** Physical and chemical composition of the cement.

Properties	Cement			
Physical	Specific gravity		3.15	
	Fineness (cm^2^/g)		3350	
Chemical (%)	CaO	63.1	C_3_S	51.0
	SiO_2_	21.7	C_2_S	25.0
	Al_2_O_3_	5.7	C_3_A	9.0
	Fe_2_O_3_	3.2	C_4_AF	9.0
	MgO	2.8	CaSO_4_	4.0
	SO_3_	2.2		

**Table 5:** Physical properties of the aggregate.

Type	Density (g/cm^3^)	Absorption (%)	Fineness Modulus
Sand	2.60	1.01	2.48
Gravel	2.68	1.35	6.76

**Table 6:** Slump value according to adding of WRA.

W/C (%)	Type	SAP Adding Ratio (C×%)				
		0 (Ref.)	0 (A.E)	1.0	1.5	2.0
40	Slump (mm)	123	124	125	130	140
	WRA (C×%)	0.4	0.4	0.8	1.0	3.0
50	Slump (mm)	155	158	148	153	150
	WRA (C×%)	0.3	0.3	0.7	0.9	2.0

**Table 7:** Pore distribution according to pore size (300 μm).

W/C (%)	Curing Condition	Pore Size (μm)	Percent of Pores (%)				
			0 (Ref.)	0 (AE)	1.0	1.5	2.0
40	Sealed	10~300	75.4	78.8	79.7	80.0	78.0
		300~	24.6	21.3	20.3	20.0	22.0
	Water	10~300	72.6	76.9	79.6	81.8	80.2
		300~	27.4	23.1	20.5	18.2	19.8
50	Sealed	10~300	72.6	75.8	77.1	76.9	82.3
		300~	27.4	24.2	23.0	23.1	17.7
	Water	10~300	76.2	78.1	79.1	81.4	83.4
		300~	23.8	21.9	20.9	18.6	16.6

## Data Availability

Not applicable.
